# Atrial resting membrane potential confers sodium current sensitivity to propafenone, flecainide and dronedarone

**DOI:** 10.1016/j.hrthm.2021.03.016

**Published:** 2021-07

**Authors:** Andrew P. Holmes, Priyanka Saxena, S. Nashitha Kabir, Christopher O’Shea, Stefan M. Kuhlmann, Suranjana Gupta, Dannie Fobian, Clara Apicella, Molly O’Reilly, Fahima Syeda, Jasmeet S. Reyat, Godfrey L. Smith, Antony J. Workman, Davor Pavlovic, Larissa Fabritz, Paulus Kirchhof

**Affiliations:** ∗ Institute of Cardiovascular Sciences, University of Birmingham, Birmingham, United Kingdom; † Institute of Clinical Sciences, University of Birmingham, Birmingham, United Kingdom; ‡ Institute of Cardiovascular & Medical Sciences, University of Glasgow, Glasgow, United Kingdom; § Department of Cardiology, Charité-Universitätsmedizin, Berlin, Germany; ¶ Computational Neurophysiology Lab, Indian Institute of Technology (IIT) Bombay, Mumbai, India; ‖ Equipe Gametes to Birth, Institut Cochin, U1016 INSERM, Paris, France; ∗∗ Department of Cardiology, University Hospital Birmingham, Birmingham, United Kingdom; †† University Heart and Vascular Center, UKE Hamburg, Hamburg, Germany; ‡‡ German Center for Cardiovascular Research (DZHK), partner site Hamburg/Kiel/Lübeck, Hamburg, Germany

**Keywords:** Atrial action potential, Atrial fibrillation, Dronedarone, Flecainide, PITX2, Propafenone, Resting membrane potential

## Abstract

**Background:**

Although atrial fibrillation ablation is increasingly used for rhythm control therapy, antiarrhythmic drugs (AADs) are commonly used, either alone or in combination with ablation. The effectiveness of AADs is highly variable. Previous work from our group suggests that alterations in atrial resting membrane potential (RMP) induced by low *Pitx2* expression could explain the variable effect of flecainide.

**Objective:**

The purpose of this study was to assess whether alterations in atrial/cardiac RMP modify the effectiveness of multiple clinically used AADs.

**Methods:**

The sodium channel blocking effects of propafenone (300 nM, 1 μM), flecainide (1 μM), and dronedarone (5 μM, 10 μM) were measured in human stem cell–derived cardiac myocytes, HEK293 expressing human Na_V_1.5, primary murine atrial cardiac myocytes, and murine hearts with reduced *Pitx2c*.

**Results:**

A more positive atrial RMP delayed I_Na_ recovery, slowed channel inactivation, and decreased peak action potential (AP) upstroke velocity. All 3 AADs displayed enhanced sodium channel block at more positive atrial RMPs. Dronedarone was the most sensitive to changes in atrial RMP. Dronedarone caused greater reductions in AP amplitude and peak AP upstroke velocity at more positive RMPs. Dronedarone evoked greater prolongation of the atrial effective refractory period and postrepolarization refractoriness in murine Langendorff-perfused *Pitx2c*^*+/–*^ hearts, which have a more positive RMP compared to wild type.

**Conclusion:**

Atrial RMP modifies the effectiveness of several clinically used AADs. Dronedarone is more sensitive to changes in atrial RMP than flecainide or propafenone. Identifying and modifying atrial RMP may offer a novel approach to enhancing the effectiveness of AADs or personalizing AAD selection.

## Graphical abstract

**Figure:**
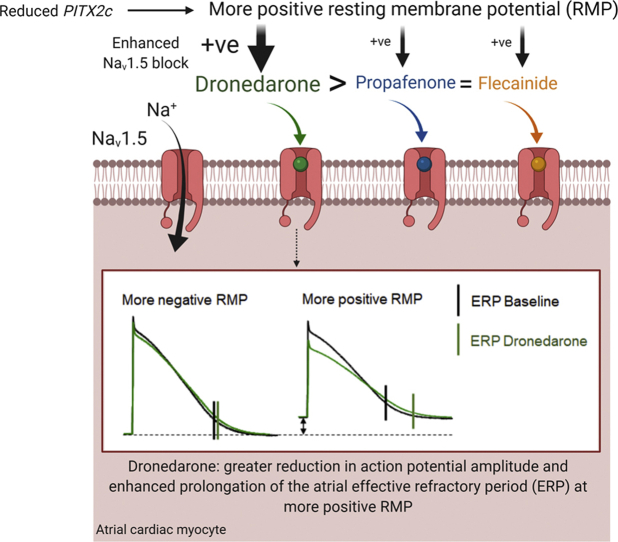

## Introduction

Rhythm control therapy is used in 10%–20% of patients with atrial fibrillation (AF) to improve AF-related symptoms, often involving antiarrhythmic drugs (AADs) as first-line therapy^1^ or in combination with AF ablation.^2^ Although anticoagulation now prevents most ischemic strokes in AF patients, the rates of stroke, heart failure, unplanned hospitalizations, and cardiovascular death remain high.^1^^,^^3^ Data suggest that restoring and maintaining sinus rhythm in patients with recently diagnosed AF provides clinical benefit compared to symptom-directed, selective rhythm control therapy.^4^ This clinical benefit was achieved using a treatment strategy comprising AADs and AF ablation.^4^ These results enhance the need for widely accessible and effective rhythm control therapy.

AADs are readily available in many cardiovascular care settings,^5^ but their effectiveness is variable.^1^ Predicting efficacy remains an unresolved clinical challenge,^1^^,^^3^ and practice patterns suggest that local protocols rather than patient factors determine AAD selection.^5^ Methods supporting the selection of effective AAD therapy could improve delivery of accessible rhythm control therapy in patients with AF.^6^

An important factor in determining AAD effectiveness likely is the underlying atrial electrical dysfunction,^6^ including a more positive atrial resting membrane potential (RMP).^7^ A shift in RMP directly modifies the efficacy of the sodium channel blocker flecainide in murine atria.^7^ Whether this effect is evident in humanized cardiac cell models has not been tested. The effect of a more positive RMP on other AADs with sodium channel blocking capabilities has not been assessed in atrial cardiac myocytes or humanized cardiac cell models. The aim of this study was to quantify and compare the impact of different RMPs on the sodium channel blocking efficacy of propafenone, flecainide, and dronedarone in human cardiac cell models, primary murine atrial cells, and clinically relevant genetic animal models.

## Methods

Details of the methods are given in the Supplemental Methods.

### Ethics and approval

Procedures and experiments involving human atrial cells were approved by the West of Scotland Research Ethics Service (REC 17/WS/0134). The human research reported in this article adhered to the Helsinki Declaration. All animal procedures were performed in accordance with UK Animals (Scientific Procedures) Act 1986 and were approved by the UK Home Office (PFDAAF77F). Animal research reported in this article adhered to the ARRIVE and Guide for the Care and Use of Laboratory Animals guidelines. Hearts were isolated from wild-type (WT) and *Pitx2c*^*+/–*^ male and female adult mice (N = 77) bred on a MF1 background, by thoracotomy under deep terminal isoflurane anesthesia (4%–5% isoflurane in O_2_, 1.5 L/min).

### Recordings of sodium currents and action potentials in human Na_V_1.5/SCN1B-expressing HEK293 cells and human-induced pluripotent stem cell–derived cardiac myocytes

For sodium currents (I_Na_), whole-cell patch-clamp recordings were obtained as described previously.^7^^,^^8^ I_Na_ was elicited in 100-ms steps to –30 mV from holding potentials of –100 to –70 mV. Currents were measured before and after propafenone (300 nM or 1 μM; Sigma-Aldrich, Gillingham, UK), dronedarone (5 and 10 μM; Sigma-Aldrich, Gillingham, UK), and flecainide (1 μM; Sigma-Aldrich, Gillingham, UK). These concentrations are consistent with those previously reported in the literature.^7^^,^^9^^,^^10^ Cells were paced at 1 Hz while the drugs were introduced to the perfusate.

Action potentials (APs) were recorded at 36°–37°C in the whole-cell current-clamp configuration and triggered by 2-ms current injections (1.5 nA). AP trains were stimulated at 1 Hz. APs were recorded at RMPs from –90 to –65 mV by varying the background current injection. Action potential amplitude (APA) and peak AP phase 0 upstroke velocity (dV·dt^-1^) were analyzed using modified algorithms from ElectroMap.^11^

### Recordings of human atrial APs

Right atrial tissue samples were obtained from 6 adult patients undergoing cardiac surgery (Supplemental Table 1). Cardiomyocytes were enzymatically isolated from this tissue using the “chunk” method.^12^ APs were stimulated and recorded at 35°–37°C by whole-cell patch clamp, using an AxoClamp 2B amplifier (Axon Instruments, Foster City, CA, USA) and WinWCP or WinEDR software (J Dempster, Strathclyde Institute of Pharmacy & Biomedical Sciences, University of Strathclyde, Glasgow, UK).

### Cell isolation and recordings of sodium currents in primary murine left atrial cardiac myocytes

Hearts were isolated from WT and *Pitx2c*^*+/–*^ mice and digested using a Langendorff apparatus as described previously.^13^ Peak I_Na_ was recorded as described for human-induced pluripotent stem cell–derived cardiac myocytes (hiPSC-CMs) and HEK293 cells, except that a low sodium external solution was used.

### Recordings of murine left atrial transmembrane APs

Transmembrane APs were recorded at 37°C from the epicardial surface of WT and *Pitx2c*^*+/–*^ left atrium (LA) using custom-made glass microelectrodes containing 3 M KCl (resistance 15–20 MΩ) as described previously.^13^^,^^14^

### Recordings of murine LA monophasic APs

LA monophasic APs were recorded at 37°C from Langendorff-perfused beating hearts isolated from WT and *Pitx2c*^*+/–*^ mice as described previously.^7^ Monophasic APs were recorded over a range of 120-, 100-, and 800-ms cycle lengths. Programmed stimulation was performed at baseline and with dronedarone (10 μM).

### Data analysis

Values are given as mean ± SEM. Single cell/heart/patient measurements are shown as individual points. Statistical analysis was performed using 1-way repeated measures analysis of variance, 2-way analysis of variance with Bonferroni *post hoc* analysis, or a paired/unpaired 2-tailed Student *t* test (Prism8, GraphPad, San Diego, CA). *P* <.05 was considered significant.

## Results

### Propafenone and dronedarone inhibit Na_V_1.5 currents more effectively at more positive RMPs

**Figure 1:**
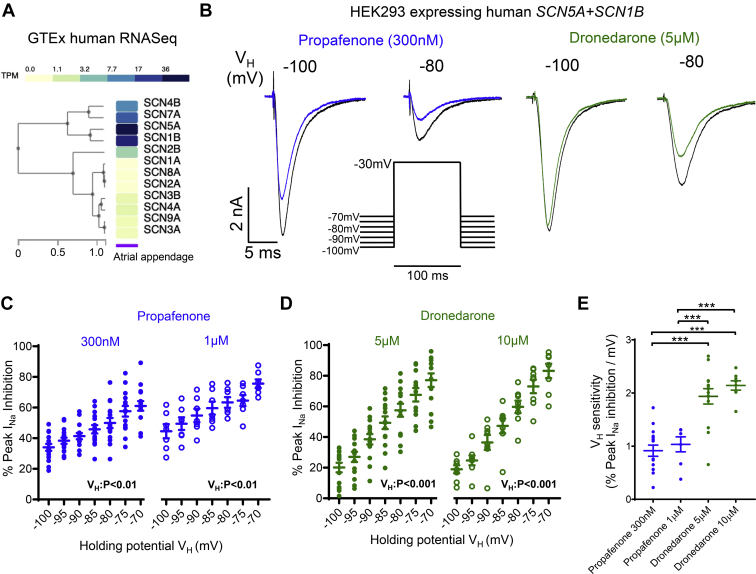
Human Na_V_1.5 sodium current (I_Na_) inhibition by propafenone and dronedarone is enhanced at more positive resting membrane potentials (RMPs). **A:** Genotype-Tissue Expression (GTEx) data showing relative mean expression of sodium channel genes in human atrial appendage (N = 429 samples). **B:** Inhibition of I_Na_ caused by propafenone (300 nM) and dronedarone (5 μM) at 2 different resting membrane potentials/holding potentials (RMP/V_H_). **C, D:** Effect of RMP/V_H_ on I_Na_ inhibition by propafenone at 300 nM (N = 15 cells) and 1 μM (N = 8 cells), and dronedarone at 5 μM (N = 14 cells) and 10 μM (N = 8 cells). Data are given as mean ± SEM, 1-way repeated measures analysis of variance (ANOVA). **E:** RMP/V_H_ sensitivities of propafenone and dronedarone. ∗∗∗*P* <.001, 2-way ANOVA with Bonferroni *post hoc* analysis.

Analysis of the Genotype-Tissue Expression (GTEx) database identified *SCN5A* and *SCN1B* as the most highly expressed alpha- and beta-subunits respectively in human atria (Figure 1A). Therefore, the effects of dronedarone and propafenone were assessed in HEK293 cells stably expressing *SCN5A* and *SCN1B*. For propafenone and dronedarone, peak I_Na_ inhibition progressively increased at more positive RMP/V_H_ (Figure 1B–1D). This was consistent at both concentrations tested for each AAD (Figure 1C and 1D). Comparison between agents showed that dronedarone has an almost 2-fold greater sensitivity (percent inhibition per mV increase in RMP/V_H_) to RMP/V_H_ than propafenone (*P* <.01) (Figure 1E). For both agents, RMP/V_H_ sensitivity was not modified by concentration (Figure 1E).

**Figure 2:**
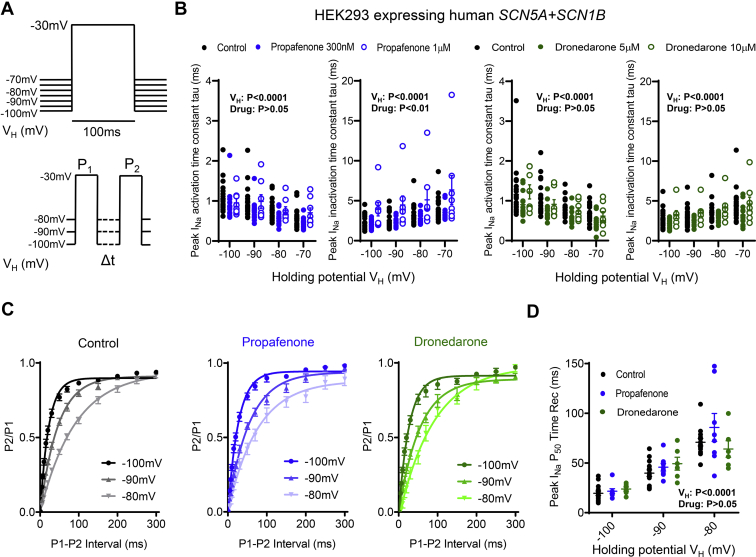
Human Na_V_1.5 sodium current (I_Na_) activation, inactivation, and time-dependent recovery kinetics are sensitive to changes in resting membrane potential (RMP). **A:** Protocols used to measure I_Na_ activation/inactivation time kinetics **(top)** and time-dependent recovery **(bottom)**. **B:** Activation and inactivation time constants (tau) measured at different resting membrane potentials/holding potentials (RMP/V_H_) for control, propafenone (300 nM and 1 μM; N = 23 cells total), and dronedarone (5 μM and 10 μM; N = 21 cells total). One-way repeated measures analysis of variance (ANOVA). **C:** I_Na_ time-dependent recovery measured in control cells (N = 14 cells), propafenone-treated cells (300 nM; N = 8 cells), and dronedarone-treated (5 μM; N = 6 cells). **D:** Effect of RMP/V_H_ on mean 50% I_Na_ recovery times (P_50_) for all 3 groups. Data are given as mean ± SEM, 2-way repeated measures ANOVA with Bonferroni *post hoc* analysis.

A more positive RMP/V_H_ decreased the peak I_Na_ activation time constant, an effect that was not further modified by propafenone or dronedarone (Figures 2A and 2B). More positive RMP/V_H_ increased the inactivation time constant, consistent with a slower rate of current decay (Figure 2B). Additionally, a more positive RMP/V_H_ significantly slowed the time-dependent peak I_Na_ recovery rate under control conditions and in the presence of propafenone (300 nM) and dronedarone (5 μM) (Figure 2C). Comparison of the 50% recovery times (P_50_) showed that neither propafenone nor dronedarone caused further slowing of recovery times at any of the RMP/V_H_ tested (Figure 2D).

### RMP modifies human atrial AP morphology

**Figure 3:**
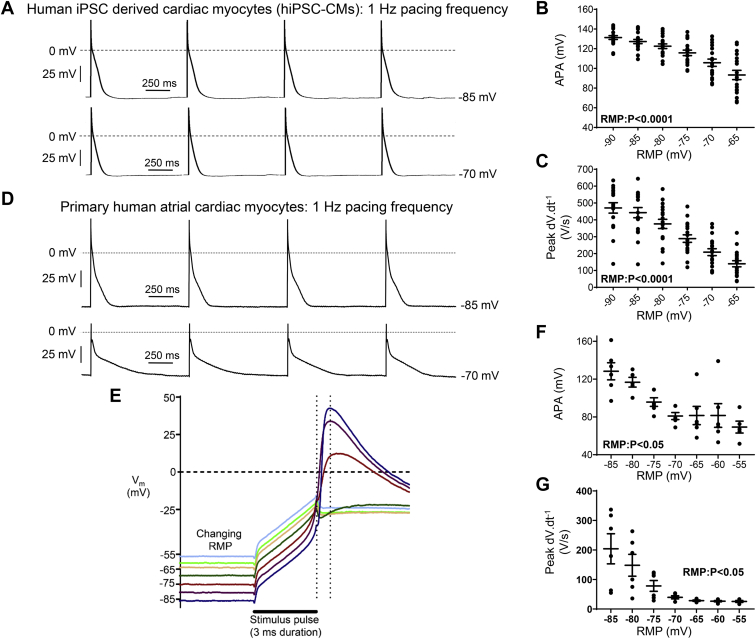
Resting membrane potential (RMP) regulates the human atrial action potential (AP). **A:** Short trains of APs measured at two different RMPs (–85 and –70 mV) in a single human-induced pluripotent stem cell–derived cardiac myocyte (hiPSC-CM), paced at 1 Hz. **B, C:** Mean data from hiPSC-CMs (N = 18 cells). **D:** Short trains of APs recorded at 2 RMPs (–85 and –70 mV) in a single primary human right atrial cardiac myocyte, paced at 1 Hz. **E:** Representative superimposed AP traces recorded from a single primary human right atrial myocyte, paced at 1 Hz. *Vertical dashed cursors* indicate the end of the stimulus and the action potential amplitude (APA), respectively. Peak dV·dt^-1^ was measured between these 2 cursors to avoid the stimulus current. **F, G:** Mean data (N = 14 cells; 6 patients). Data are given as mean ± SEM, 1-way analysis of variance.

In hiPSC-CMs paced at 1 Hz, at more positive RMPs (incrementing from –90 to –65 mV) there was a graded reduction in both APA and peak dV·dt^–1^ (Figures 3A–3C). In primary human atrial myocytes paced at 1 Hz, current-clamping of RMP in 5-mV steps from –85 to –55 mV evoked a marked decrease in peak dV·dt^-1^, from ∼205 V/s at RMP –85 V, to ∼25 V/s at RMPs of –65 mV or more positive (Figures 3D–3G). This is consistent with voltage-dependent inactivation of I_Na_ between –70 and –60 mV and with the corresponding decrease in APA (Figures 3D–3G).

### Dronedarone causes greater changes in AP morphology at more positive RMPs in hiPSC-CMs

**Figure 4:**
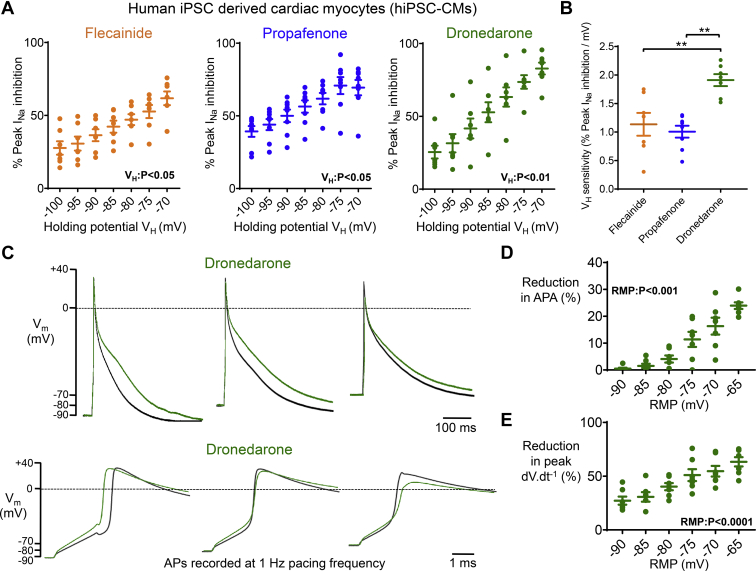
Dronedarone causes greater inhibition of cardiac action potential amplitude (APA) and upstroke velocity at more positive resting membrane potentials (RMPs). **A:** Data from human-induced pluripotent stem cells–derived cardiac myocytes (hiPSC-CMs) demonstrating the effect of resting membrane potential/holding potential (RMP/V_H_) on sodium current (I_Na_) inhibition by flecainide (1 μM; N = 7 cells), propafenone (300 nM; N = 8 cells) and dronedarone (5 μM; N = 7 cells). **B:** RMP/V_H_ sensitivities of flecainide, propafenone, and dronedarone. ∗∗*P* <.01, 1-way analysis of variance (ANOVA) with Bonferroni *post hoc* analysis. **C:** Action potentials (APs) measured at 3 different RMPs (–100, –90, and –70 mV) measured in a single hiPSC-CM ± dronedarone (5 μM), paced at 1 Hz. **D, E:** Mean data from hiPSC-CMs (N = 7 cells). Data are given as mean ± SEM, 1-way repeated measures ANOVA.

In hiPSC-CMs, control peak I_Na_ was progressively reduced as RMP/V_H_ became more positive (from –5.5 ± 0.5 nA at –90 mV, to –0.8 ± 0.2 nA at –70 mV; n = 15 cells; *P* <.001). For propafenone (300 nM) and dronedarone (5 μM), I_Na_ inhibition was enhanced at more positive RMP/V_H_ (Figure 4A). RMP/V_H_ sensitivity of propafenone (∼1%/mV) was similar to that of flecainide (1 μM) (Figure 4B). Dronedarone inhibition of peak I_Na_ was 2-fold more sensitive to changes in RMP (∼2% per mV) compared to both propafenone and flecainide (Figure 4B).

Because dronedarone showed the highest sensitivity to RMP/V_H_, this agent was taken forward for functional testing on hiPSC-CMs. Dronedarone caused action potential duration (APD) prolongation in the majority of cells, consistent with its known inhibition of I_Kr_, and a reduction in both APA and peak dV·dt^-1^ (Figure 4C–4E, and Supplemental Figures 1 and 2). The magnitude of responses to dronedarone varied at different RMPs. At RMP of –90 mV, dronedarone caused a minimal reduction in APA (Figure 4D, and Supplemental Figures 1 and 2). However, dronedarone caused a greater reduction in APA as RMP became progressively more positive (Figure 4D, and Supplemental Figures 1 and 2). Similarly, dronedarone more effectively decreased peak dV·dt^-1^ at more positive RMPs (Figure 4E).

### Dronedarone causes greater effective refractory period prolongation and postrepolarization refractoriness in atria with reduced *Pitx2c*

*Pitx2c*^*+/–*^ LA cardiac myocytes had a more positive RMP and reduced APA (Supplemental Figures 3A and 3B), as expected.^7^ Peak I_Na_ was not different between genotypes at fixed V_H_/RMP (Supplemental Figure 3C). Murine LA I_Na_ inhibition by dronedarone, propafenone, and flecainide was enhanced at more positive RMP/V_H_ and was similar for both genotypes at fixed V_H_/RMPs (Supplemental Figures 3D and 3E).

**Figure 5:**
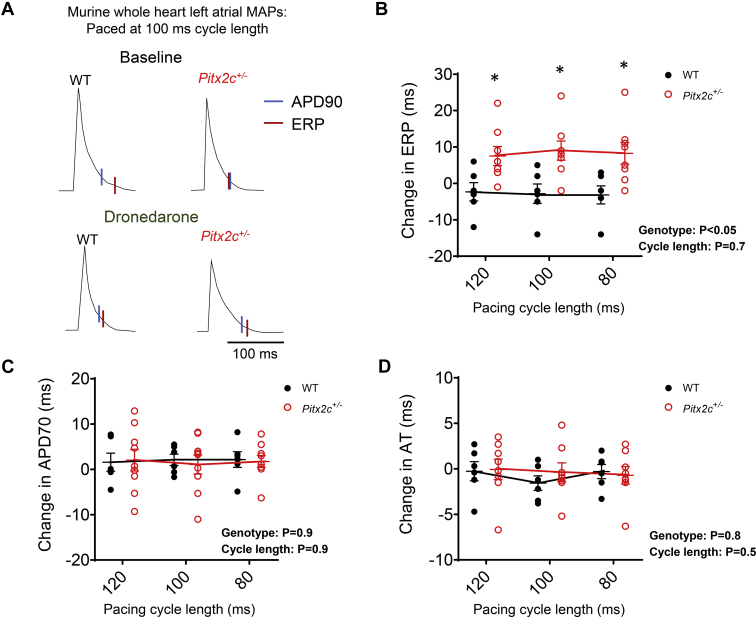
Dronedarone causes greater effective refractory period (ERP) prolongation in murine left atria (LA) with reduced *Pitx2c* compared to wild type (WT). **A:** Monophasic action potentials (MAPs) recorded at 100-ms pacing cycle length from WT and *Pitx2c*^*+/–*^ intact LA. Action potential duration at 90% repolarization (APD90; *blue line*) and ERP (*red line*) before and after dronedarone (10 μM) are indicated. **B–D:** Mean effects of dronedarone on LA ERP **(B)**, action potential duration at 70% repolarization (APD70) **(C)**, and activation time (AT) in WT (N = 6 hearts; 6 animals) and *Pitx2c*^*+/–*^ (N = 9 hearts; 9 animals), measured at pacing cycle lengths at 120, 100, and 80 ms **(D)**. Data are given as mean ± SEM. ∗*P* <.05 WT vs *Pitx2c*^*+/–*^, 2-way analysis of variance with Bonferroni *post hoc* analysis.

**Figure 6:**
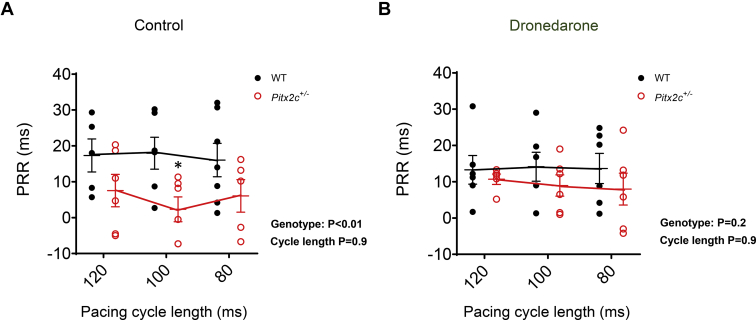
Dronedarone reverses the shortening of postrepolarization refractoriness (PRR) in murine left atria with reduced *Pitx2c.* Data show PRR before **(A)** and after **(B)** dronedarone (10 μM) in wildtype (WT; N = 6 hearts; 6 animals) and *Pitx2c*^*+/–*^ (N = 6 hearts; 6 animals), measured at pacing cycle lengths of 120, 100, and 80 ms. Data are given as mean ± SEM. ∗*P* <.05 WT vs *Pitx2c*^*+/–*^, 2-way analysis of variance with Bonferroni *post hoc* analysis.

In isolated, beating mouse hearts, dronedarone (10 μM) caused a greater prolongation of the LA effective refractory period (ERP) in *Pitx2c*^*+/–*^ than in WT littermates (Figures 5A and 5B, and Supplemental Figure 4). Dronedarone prolonged APD similarly in both genotypes, and there were no genotype-dependent differences in activation times (ATs) (Figures 5C and 5D). Postrepolarization refractoriness (PRR) (ERP-APD_90_) was significantly shorter at baseline in *Pitx2c*^*+/–*^ LA (Figure 6A, and Supplemental Table 2). This effect was abolished after treatment with dronedarone (Figure 6B) as dronedarone caused greater prolongation of PRR in *Pitx2c*^*+/–*^ LA (Figure 6 and Supplemental Table 2).

## Discussion

### Main findings

A more positive RMP increases the effectiveness of propafenone, flecainide, and dronedarone in human cardiac cell models and primary murine atrial cardiac myocytes. Dronedarone is more sensitive to RMP than propafenone or flecainide. Dronedarone-induced PRR is enhanced in atria, with a more positive RMP caused by a reduction in *Pitx2c*. Selecting dronedarone or other sodium channel blockers based on markers for a more positive RMP and/or as potential companion therapies with RMP-depolarizing agents in patients with a hyperpolarized RMP has emerged as a promising approach to improve the effectiveness of AAD therapy for AF.

### Atrial RMP modulates the effectiveness of AADs

Approximately 10%–20% of AF patients currently receive rhythm control therapy to improve symptoms.^1^ This proportion may increase due to the clinical benefit of early rhythm control therapy,^4^ enhancing the need for readily available and effective rhythm control therapy. Although some patients respond well to AAD therapy, many experience early recurrences, with symptomatic AF recurrence occurring in 40%–70% of AF patients within 6–12 months.^1^ Three of the most used AADs inhibit the cardiac sodium channel and were tested here: flecainide, propafenone, and dronedarone.

The present study demonstrated that RMP modulates the effectiveness of 3 AADs in human cardiac cell models and murine LA cells. AF has been associated with diverse changes in atrial RMP, including a more negative RMP (∼5–10 mV).^12^^,^^15^ Other studies suggest an unchanged^16^ or more positive RMP in AF,^17^ illustrating the heterogeneous electrical changes in AF. Variations in different K^+^ currents, including I_K1_, I_KACh_, I_KCa_, and I_Kleak_,^18^ can explain these findings. In instances in which RMP is altered, our findings suggest that the extent of this modification may be a contributing factor to the overall efficacy of flecainide, propafenone, and dronedarone.

Previous studies have shown that dronedarone is more effective at blocking I_Na_ at more positive RMPs in guinea pig ventricular cardiac myocytes.^9^ Our data show that this effect is consistent in hiPSC-CMs and murine atrial cells. One reason for the observed atrial selectivity of dronedarone likely is the more positive RMP in comparison with ventricular myocytes.^9^ Our data also show that dronedarone is more sensitive to RMP than both propafenone and flecainide. The reduced RMP sensitivity of flecainide and propafenone in comparison to dronedarone could be a contributing factor to their apparent lack of differential atrial/ventricular selectivity.^19^ Other biophysiological differences between atrial and ventricular myocytes may modify drug sensitivity, such as alterations in accessory subunits, ion channel localization, and posttranslational modifications. Although *Pitx2* is expressed in all chambers in the developing heart, *Pitx2* in the adult heart is almost completely confined to the LA.^20^^,^^21^ Although it may be tempting to speculate that the difference in RMP between LA and left ventricular cardiomyocytes is due to differential *Pitx2* expression, other mechanisms must be at play, as right atrial cardiomyocytes also have a more positive RMP compared to ventricular cardiomyocytes. Our experiments in *Pitx2*-deficient atria demonstrated that the effect of RMP on dronedarone leads to increased PRR, an important mechanism of AADs.^22^

### Is there a link between a more positive atrial RMP and the genomic markers for AF on chromosome 4q25?

Key single nucleotide polymorphisms on chromosome 4q25, adjacent to the *PITX2* gene, are by far the strongest genomic signal associated with AF.^23^ *PITX2* is an important transcription factor that regulates ion channel expression and electrical integrity in the adult LA.^24^ The single nucleotide polymorphisms lie in a region that has putative transcriptional enhancer activity of *PITX2.*^25^^,^^26^ Risk alleles on chromosome 4q25^27^ and reduced LA *PITX2* expression^28^ are associated with recurrent AF on rhythm control therapy. In this study, we demonstrated that murine cardiac myocytes with low *Pitx2c* mRNA expression have a more positive RMP, consistent with previous reports,^7^^,^^24^ suggesting one potential mechanism for this clinical effect. Dronedarone caused greater ERP prolongation in *Pitx2c*^*+/–*^ atria than in WT, leading to enhanced atrial PRR. However, dronedarone did not cause greater APD elongation in either genotype. Therefore, we do not believe that the exaggerated increase of ERP in *Pitx2c*^*+/–*^–deficient atria is due to excessive lengthening of the APD but instead is dependent on an enhanced level of sodium channel block. This is consistent with observations from our human and animal cell experiments in which sodium channel block was elevated at more positive RMPs. It also demonstrates that small fluctuations in RMP can have important functional effects on AAD efficacy. It has been reported that flecainide extends the ERP more in *Pitx2c*^*+/–*^ hearts compared to WT, whereas sotalol has no genotype-dependent effects.^7^ Collectively, these data suggest that both dronedarone and flecainide may provide effective treatment in atria with reduced *PITX2*. To extend these findings, a key next step will be to determine whether human atrial cells with reduced *PITX2* have a more positive RMP and whether they respond well to flecainide and/or dronedarone.

### Clinical perspective

Currently no clinically accessible marker identifies patients with a depolarized atrial RMP, although elevated bone morphogenic protein 10 has recently been suggested as a blood biomarker for reduced LA *PITX2.*^28^ Drugs that depolarize atrial RMP could be combined with sodium channel blockers such as dronedarone to enhance their effectiveness. More research is required to better understand how RMP is altered by different genetic or environmental causes of AF, and clinical markers identifying patients with a depolarized atrial RMP are needed to develop and test such therapeutic concepts.

### Study limitations

This study utilized murine atrial cardiac myocytes, hiPSC-CMs, and HEK293 expressing human cardiac sodium channels. We also provided validation in perfused beating murine atria (Figures 5 and 6). However, we did not perform differentiation protocols to generate atrial-specific hiPSC-CMs. Validation of the impact of atrial RMP on AAD sensitivities and electrical function specifically in primary human atrial tissue is still warranted. There are some limitations with using hiPSC-CMs due to their relatively immature status.^29^ However, the study findings showed consistency with both primary murine atrial cardiac myocytes and HEK293 cells expressing human cardiac *Scn5a*. Whether atrial RMP is stable over time and/or whether regional variability in the RMP affect atrial function and recurrent AF should be studied in future projects. Although enhanced PRR and suppression of automatic activity are valid surrogates for AAD action, this study could not test whether RMP had an effect on spontaneous or inducible AF. This needs to be addressed in future studies, ideally investigating patients and using clinical markers for patients with a more positive atrial RMP. The range of drug concentrations used in this study are consistent with values reported in previous experimental literature,^7^^,^^9^^,^^10^ are within the range reported to inhibit I_Na_, and are either within or close to the reported therapeutic plasma concentrations. As exact intra-atrial concentrations have not been reported, we cannot say for certain that the concentrations used in this study precisely mimic those present *in vivo* at the level of a single atrial cardiac myocyte.

## Conclusion

Dronedarone, propafenone, and flecainide all are sensitive to changes in atrial RMP, promoting greater peak I_Na_ inhibition at more positive RMP. Dronedarone is most sensitive to variations in RMP. In human cardiac cell models, dronedarone reduces APA and peak dV·dt^-1^ to a greater extent at more positive RMPs. Dronedarone causes greater prolongation of ERP and PRR in *Pitx2c*^*+/–*^ atria that have a more positive RMP compared to WT. Therefore, RMP is an important modulator of the I_Na_ inhibiting effect of different clinically used AADs. Targeting RMP to increase the effectiveness of AADs or selecting AADs based on specific RMP alterations in different types of AF provide novel approaches for improving patient outcomes.
