# Supplementary material for: Atrial resting membrane potential confers sodium current sensitivity to propafenone, flecainide and dronedarone
Source: Heart Rhythm. 2021 Jul;18(7):1212–20. doi: 10.1016/j.hrthm.2021.03.016 (PMC8259123; doi:10.1016/j.hrthm.2021.03.016)
Supplement: Supplementary Material [file mmc1.pdf]

Full Methods

Ethics and Approval

Procedures and experiments involving human atrial cells were approved by the West of Scotland Research Ethics Service (REC: 17/WS/0134). Written, informed consent was obtained from all patients prior to inclusion in this study. All procedures were performed in accordance with the Declaration of Helsinki. All animal procedures were performed in accordance with UK Animals (Scientific Procedures) Act 1986, and approved by the UK Home Office (PPL PFDAAF77F) and locally by the Animal Welfare and Ethical Review Body (AWERB) at University of Birmingham. The animal research reported in this paper adhered to the ARRIVE and Guide for the Care and Use of Laboratory Animals guidelines. Hearts were isolated from male and female adult mice (N=77 in total, 3-5 month old) bred on a MF1 background (back-crossed more than 10 generations). Murine hearts were extracted by thoracotomy under inhalation isoflurane anaesthesia (4-5% isoflurane in O_2, 1.5L.min^{-1}). Removal of the heart led to death by exsanguination. Sufficient depth of surgical anaesthesia was determined by complete absence of the pedal withdrawal reflex. *Pitx2c*$^{+/-}$ mice were generated as previously described by replacing the 4th exon, specific to *Pitx2c* isoform, neomycin selection cassette[1]. Animals were housed in individually ventilated cages under standard conditions: 12:12 hour light:dark cycle, 22 °C and 55% humidity. Food and water were available *ad libitum*.

Cell culture of human *SCN5A/SCN1B* expressing HEK293 cells and human induced pluripotent stem cells derived cardiac myocytes (hiPSC-CMs)

HEK293 cells stably expressing the human *SCN5A* and *SCN1B* (HEK293-hNav1.5) were obtained from SB Drug Discovery (SB Drug Discovery, Glasgow, UK). The HEK293-hNav1.5 cells were grown according to manufacturer's instruction. In brief, HEK293-hNav1.5 cells were plated and grown in 75 cm^2 culture flasks (Greiner Bio-One #658175) in complete medium:MEM (Sigma M5650) supplemented with L-Glutamine (Sigma G7513) final concentration 2mM, 10% Heat-inactivated FBS (Invitrogen 10500-064), Pen/Strep (Gibco 15070-063), Geneticin (Invitrogen 10131-027) 600 µg/ml, Blasticidin (Invitrogen ANT-BL-5) 4 µg/ml. For patch clamp, cells were seeded 48 or 72 hours before experiment at a density of $3.2x10^6$ and $2.1x10^6$ respectively, in T75 cm^2 flask in complete medium. Briefly, cells were gently detached with 1.5 ml of TrypLE Express (Invitrogen #12605-026), incubated at 37 °C for exactly 4 min, resuspended in EX-CELL ACF CHO Medium (Sigma #C5467) supplemented with L-Glutamine (final concentration 8 mM), to a final density of 40,000 cells/ml. Cells in suspension were transferred to glass conical flasks and incubated in shaker at 37 °C, 95 rpm until experiment. Human induced pluripotent stem cell derived cardiac myocytes (hiPSC-CMs) (iCell Cardiomyocytes[2], Cellular Dynamics, Madison, WI, USA) were thawed, plated and maintained according to manufacturer's instructions. Briefly, hiPSC-CMs were plated in plating medium (iCell Cardiomyocytes Plating Medium) at previously gelatine (0.1 %) coated 12-well plates and incubated at 37°C, 5% CO_2 . After 4-hrs the medium was exchanged with maintenance media (iCell Cardiomyocytes Maintenance Medium). The media was exchanged every 48 hrs for 7 days (timepoint of which the hiPSC-CMs display beating morphology according to manufacturer's instructions). The hiPSC-CMs were released using EDTA-Trypsin (0.05%) and suspended in 5 mL maintenance medium. Cells in suspension were

transferred to glass conical flasks and incubated in shaker at 37 °C, 95 rpm until experimentation.

Recordings of sodium currents and action potentials in hNav1.5/SCN1B expressing HEK293 cells and hiPSC-CMs

To record sodium currents (I_{Na}), HEK293-hNav1.5 or hiPSC-CMs were perfused at 3ml.min^{-1}, 22±0.5 °C, with a solution containing in (mM): NaCl (140), KCl (4.5), HEPES (10), $CaCl_2$ (1.8), $MgCl_2$ (1.2) and glucose (10), pH 7.4. 2mM $NiCl_2$ was used to block L-type Ca^{2+} currents. Whole cell patch clamp recordings were obtained in voltage clamp mode using borosilicate glass pipettes (tip resistance 1.5–3 MΩ) as described previously[2, 3]. The internal pipette solution contained in (mM): CsCl (115), NaCl (5), HEPES (10), EGTA (10), MgATP (5), $MgCl_2$ (0.5) and TEA (20), pH 7.2 (CsOH). The pipette offset potential was corrected before seal formation. I_{Na} was elicited at 100ms steps to -30mV from holding potentials of -100 to -70mV (in 5mV increments). At least 5 sodium currents were recorded at each potential to achieve steady-state. Currents were stimulated at 1Hz. Currents were measured before and after propafenone (Sigma, 300nM or 1μM), dronedarone (Sigma, 5 and 10μM) and flecainide (Sigma, 1μM); clinically reported concentrations of these agents[4]. The liquid junction potential (LJP) was between 2-3mV and was not corrected. Series resistance was compensated between 80 and 100% and experiments were terminated if series resistance abruptly increased (>20%) or achieved a value more than 10MΩ. Activation and inactivation time constants were calculated by fitting the respective phases to single exponential functions[5]. By definition, exponential growth and decay are governed by equations [1] and [2], respectively. Here, I is the value attained at time t,

$\tau\ (tau)$ is the time constant of the respective phases, and I_{max} is the peak when $t \to \infty$ [1] or when $t = 0$ [2].

$$I = I_{max} * \left(1 - e^{-t/\tau_{rise}}\right) \qquad [1]$$

$$I = I_{max} * e^{-t/\tau_{fall}} \qquad [2]$$

Time dependent peak I_{Na} recovery kinetics were evaluated using 20ms P1-P2 pulse protocols with increasing time delay ranging from 1 to 300ms. These experiments were performed at 3 different holding potentials (V_H) (-100mV, -90mV and -80mV), using a test potential of -30mV.

To record action potentials (APs), hiPSCs-CMs that had been cultured in a beating monolayer were initially dissociated and adhered onto laminin (from Engelbreth-Holm-Swarm murine sarcoma basement membrane, Sigma, UK) coated coverslips. hiPSC-CMs were superfused at 3ml.min-1, 36-37 °C, with a solution containing in (mM): NaCl (140), KCl (5.4), HEPES (10), $CaCl_2$ (1.8), $MgCl_2$ (1.2) and glucose (10), pH 7.4. The internal pipette solution contained in (mM): K-glutamate (130), KCl (10), NaCl (10), $MgCl_2$ (0.5), MgATP (5), HEPES (10) and Na_3GTP (0.5), pH 7.2. APs were recorded in the whole cell current clamp configuration and triggered by 2ms current injections (1.5nA amplitude). This was done using an Axopatch 200B Amplifier (Molecular Devices, CA, USA) and a CED micro 1401 analogue to digital converter programmed using Signal v6 software (Cambridge Electronic Design, Cambridge, UK). APs trains were stimulated at 1Hz. APs were recorded over a physiological range of RMPs (-90 to -65mV, 5mV increments) by progressively varying the background hyperpolarising current (hyper-I) injection in the same cell. At least 10-15 cycles per RMP were

performed before measurements to allow for the AP to achieve a steady state morphology. APs were recorded before and after dronedarone (Sigma, 10μM). The measured LJP was significantly greater in these experiments (*ca* +15mV). The LJP was adjusted for in the protocol and the AP measurements were corrected offline. For example, for the LJP of +15mV to achieve a true RMP of -80mV, the holding current was adjusted to a recorded RMP of -65mV. Action potential amplitude (APA) and peak AP phase 0 upstroke velocity ($dV.dt^{-1}$) were analysed using adapted algorithms based on those published in ElectroMap[6].

Recordings of human atrial action potentials

Right atrial tissue samples were obtained from 6 adult patients (66±3 years; 4 male/2 female) undergoing cardiac surgery (67% coronary artery bypass graft, 17% aortic valve replacement, 17% mitral valve replacement). Patient characteristics are described in Supplementary Table 1. Cardiomyocytes were enzymatically isolated from these tissues using the 'chunk' method[7], stored (≤9 hr, ~20°C) in cardioplegic solution (mM): KOH (70), KCl (40), L-glutamic acid (50), taurine (20), KH_2PO_4 (20), $MgCl_2$ (3), glucose (10), HEPES (10), EGTA (0.5), pH 7.2.

Myocytes were superfused (35-37°C) with a physiological salt solution containing (mM): NaCl (140), KCl (4), $CaCl_2$ (1.8), $MgCl_2$ (1), glucose (11) and HEPES (10); pH 7.4. Microelectrodes contained (mM): K-aspartate (130), KCl (15), NaCl (10), $MgCl_2$ (1), HEPES (10), EGTA (0.1); pH 7.25. A LJP (+9 mV; bath relative to pipette) was compensated for prior to seal formation. APs were stimulated and recorded by whole-cell-patch clamp, with an Axon Instruments AxoClamp 2B amplifier (fast voltage-follower, for accurate peak $dV.dt^{-1}$ measurement) and WinWCP or WinEDR software

(written by J Dempster, Strathclyde University). Trains of APs were stimulated at 1 Hz by current-clamping with 3-ms pulses of 1.5-2 x threshold amplitude. The value of the RMP was set, and incremented in 5 mV steps, by current-clamping in bridge-mode. Specifically, the RMP was adjusted over the range of -85 to -55 mV by injecting a progressively increasing hyper-I to the same cell, as described previously[8]. Only cells in which hyper-I was < 1.5 pA/pF to achieve an RMP of -80mV were included[8]. At least 20 cycles per RMP were performed before measurements to allow for AP stabilisation.

Cell isolation and recordings of sodium currents in primary murine left atrial cardiac myocytes

Hearts were isolated from wild type (WT) and *Pitx2c*$^{+/-}$ mice and digested using HEPES-buffered Tyrode's solution containing in (mM): NaCl (140), KCl (5.4), $CaCl_2$ (1.8), $MgSO_4$ (0.8), Na_2HPO_4 (0.33, HEPES (5), glucose (11), taurine (20), $CaCl_2$ (0.03), 0.1% BSA and 20µg/mL Liberase™ (Roche, Indianapolis, IN, USA), via a vertical Langendorff apparatus[9]. Digestion time was 5-7 minutes. Flow rate was kept at 4ml min^{-1}. The solution was equilibrated with 100% O_2, heated to 37°C and had a pH of 7.4 (NaOH). Hearts were removed from the Langendorff and immediately perfused with 5ml of modified Kraft-Bruhe (KB) solution containing in (mM): DL-potassium aspartate (10), L-potassium glutamate (100), KCl (25), KH_2PO_4 (10), $MgSO_4$ (2), taurine (20), creatine (5), EGTA (0.5), HEPES (5), 0.1% BSA, and glucose 20 (pH 7.2, KOH). The LA was dissected free and cardiac myocytes were dissociated by gentle trituration with fire-polished glass pipettes (2 to 0.5mm diameter). Cells were suspended in 2ml KB buffer and Ca^{2+} was slowly reintroduced incrementally over a period of 2 hours to reach a final concentration of 1.8mM. All experiments were performed within 6-8 hours of isolation. Peak I_{Na} was recorded at different holding

potentials (V_H: -100 to -70 mV) as described for hiPSC-CMs and HEK293 cells except that a low sodium external solution was used (10mM $NaCl_2$, balanced with 130mM $C_5H_{14}ClNO$) to restrict maximum current amplitude to *ca* 4nA at -100mV V_H.

Recordings of murine left atrial transmembrane action potentials

Isolated intact LA preparations from WT and *Pitx2c*$^{+/-}$ adult mice were continuously superfused at 10 ml min^{-1} with a bicarbonate buffered Krebs-Henseleit (KH) solution containing in (mM): NaCl (118); $NaHCO_3$ (25); KH_2PO_4 (1.2); Glucose (11); $MgSO_4$ (0.8); $CaCl_2$ (1.8); KCl (3.5), equilibrated with 95%O_2/5%CO_2, pH 7.4 and heated to 37°C. Preparations were paced at 3Hz via bipolar platinum electrodes. Transmembrane action potentials (TAPs) were recorded from the central epicardial surface of the LA using custom made glass floating microelectrodes containing 3M KCl, (resistance 15-20 MΩ) as described previously[9, 10]. The RMP, APA and peak dV.dt^{-1} were analysed using a custom algorithm developed in Spike2 (CED).

Recordings of murine left atrial monophasic action potentials

LA epicardial monophasic action potentials (MAPs) were recorded from Langendorff-perfused beating hearts isolated from WT and *Pitx2c*$^{+/-}$ mice as described[3]. The perfusate solution contained in (mM): NaCl (118); $NaHCO_3$ (25); KH_2PO_4 (1.2); Glucose (11); $MgSO_4$ (0.8); $CaCl_2$ (1.8); KCl (3.5), equilibrated with 95%O_2/5%CO_2, pH 7.4, 37°C. Programmed stimulation was performed at baseline and with dronedarone (Sigma, 10µM). Preparations were paced (120, 100 and 80ms cycle lengths) from the right atrium (RA) using a 2.0 French octopolar mouse electrophysiological catheter with electrodes sized 0.5mm and spaced at 0.5mm (CIB'ERMOUSE, NuMED,LLC., N.Y., USA). Stable LA MAP recordings were obtained

using a miniaturised MAP catheter mounted on spring-loaded electrode holder. Voltage signals were amplified, digitised and viewed on a PC loaded with iox2 software (EMKA, Paris, France). The effective refractory period (ERP) was determined by using single RA extrastimuli after a train of 8 x S1 pacing, in 1ms decrements[3]. Post-repolarisation refractoriness (PRR) was defined as difference between action potential duration (APD) at 90% repolarisation and the ERP (ERP-APD90). The activation time (AT) was defined as the time from the stimulus pulse applied through the octopolar catheter in the RA to the peak upstroke of the MAP recorded in the LA.

Data Analysis.

Values in text are expressed as mean ± SEM. Single cell/heart/patient measurements are shown as individual points. Statistical analysis was performed using 1) one-way repeated measures Analysis of Variance (ANOVA), 2) a two-way ANOVA with Bonferroni post hoc analysis or 3) a paired/unpaired 2-tailed student's t-test, (Prism8, GraphPad, Cal, USA). Significance was taken as $P<0.05$.

Supplementary Tables and Figures

**Patient number**	1	2	3	4	5	6	**Averages**
Age (years)	70	76	54	57	69	70	66±3
Sex (M/F)	F	F	M	M	M	M	67% M
**Cardiac rhythm**							
Day of operation	SR	SR	SR	SR	SR	SR	100% SR
Pre-op history	SR	SR	SR	SR	pAF	SR	83% SR, 17% pAF
**Operation**							
CABG	N	N	Y	Y	Y	Y	67%
AVR	Y	N	N	N	N	N	17%
MVR	N	Y	N	N	N	N	17%
**Medication**							
$Beta_1$-antagonist	N	Y	Y	Y	Y	Y	83%
ACEI/ARB	N	N	N	Y	N	Y	33%
CCB	N	N	N	Y	Y	N	33%
Digoxin	N	N	N	N	Y	N	17%
Nicorandil	N	N	N	Y	N	N	17%
Nitrate	Y	Y	Y	Y	Y	Y	100%
Statin	Y	Y	Y	Y	Y	Y	100%
**Disease**							
Angina	N	N	Y	Y	Y	Y	67%
History of MI	N	Y	N	N	Y	Y	50%
History of HT	N	N	N	Y	Y	Y	50%
Diabetes	N	N	N	N	N	Y	17%
LVEF (%)	62	67	60	68	63	48	61±3%

Supplementary Table 1: Patients' clinical characteristics

SR=sinus rhythm; pAF=paroxysmal AF; CABG=coronary artery bypass graft surgery; AVR=aortic valve replacement; MVR=mitral valve replacement; ACEI/ARB=angiotensin converting enzyme inhibitor or angiotensin receptor blocker; CCB=calcium channel blocker;

MI=myocardial infarction; HT=hypertension; LVEF=left ventricular ejection fraction (from echocardiogram).

	Wildtype						*Pitx2c*$^{+/-}$					
**Paced CL (ms)**	120		100		80		120		100		80	
	Control	Dron	Control	Dron	Control	Dron	Control	Dron	Control	Dron	Control	Dron
**AERP (ms)**	39±4 (6)	37±5 (6)	39±4 (6)	36±6 (6)	40±5 (6)	37±6 (6)	35±4 (8)	42±4$^{\#}$ (8)	32±4 (8)	41±4$^{\#\#}$ (8)	32±4 (8)	40±3$^{\#}$ (8)
**AT (ms)**	19±2 (6)	18±2 (6)	20±3 (6)	19±2 (6)	22±4 (6)	21±4 (6)	16±1 (8)	16±1 (8)	15±1 (8)	15±1 (8)	17±1 (8)	16±1 (8)
**APD30 (ms)**	6±0.5 (6)	7±1 (6)	5±0.9 (6)	8±1 (6)	7±0.9 (6)	8±0.9 (6)	6±0.5 (9)	8±0.8 (9)	7±0.6 (9)	8±0.6 (9)	7±0.6 (9)	7±0.4 (9)
**APD50 (ms)**	10±0.6 (6)	11±2 (6)	9±1 (6)	11±1 (6)	10±0.9 (6)	11±1 (6)	12±1 (9)	16±2 (9)	13±0.9 (9)	15±1 (9)	11±0.9 (9)	13±0.7 (9)
**APD70 (ms)**	16±1 (6)	17±3 (6)	15±2 (6)	17±2 (6)	15±1 (6)	17±2 (6)	22±2 (9)	23±2 (9)	23±1** (9)	24±2* (9)	19±1 (9)	21±1 (9)
**APD90 (ms)**	23±2 (6)	26±3 (7)	23±2 (6)	27±3 (6)	25±2 (7)	25±3 (6)	30±1 (6)	36±4* (5)	33±1* (6)	35±2 (6)	31±2 (5)	33±2 (6)
**PRR (ms)**	17±5 (5)	14±5 (6)	18±5 (6)	17±4 (6)	16±5 (7)	14±4 (6)	8±4 (6)	11±1 (5)	2±3* (6)	9±3 (6)	6±4 (5)	8±4 (6)

Supplementary Table 2. Left atrial (LA) monophasic action potential (MAP) characteristics in Wildtype and *Pitx2c*$^{+/-}$ hearts ± Dronedarone (Dron; 10μM). CL - Cycle length, AERP - Atrial effective refractory period, AT - Activation time, APD30-90 - Action potential duration at 30 to 90% repolarisation, PRR - Post repolarisation refractoriness. *, ** P<0.05 and P<0.01 vs Wildtype respectively. #, ## P<0.05 and P<0.01 vs Control respectively. Two-way ANOVA with Bonferroni post-hoc analysis. Numbers in brackets denote number of hearts.

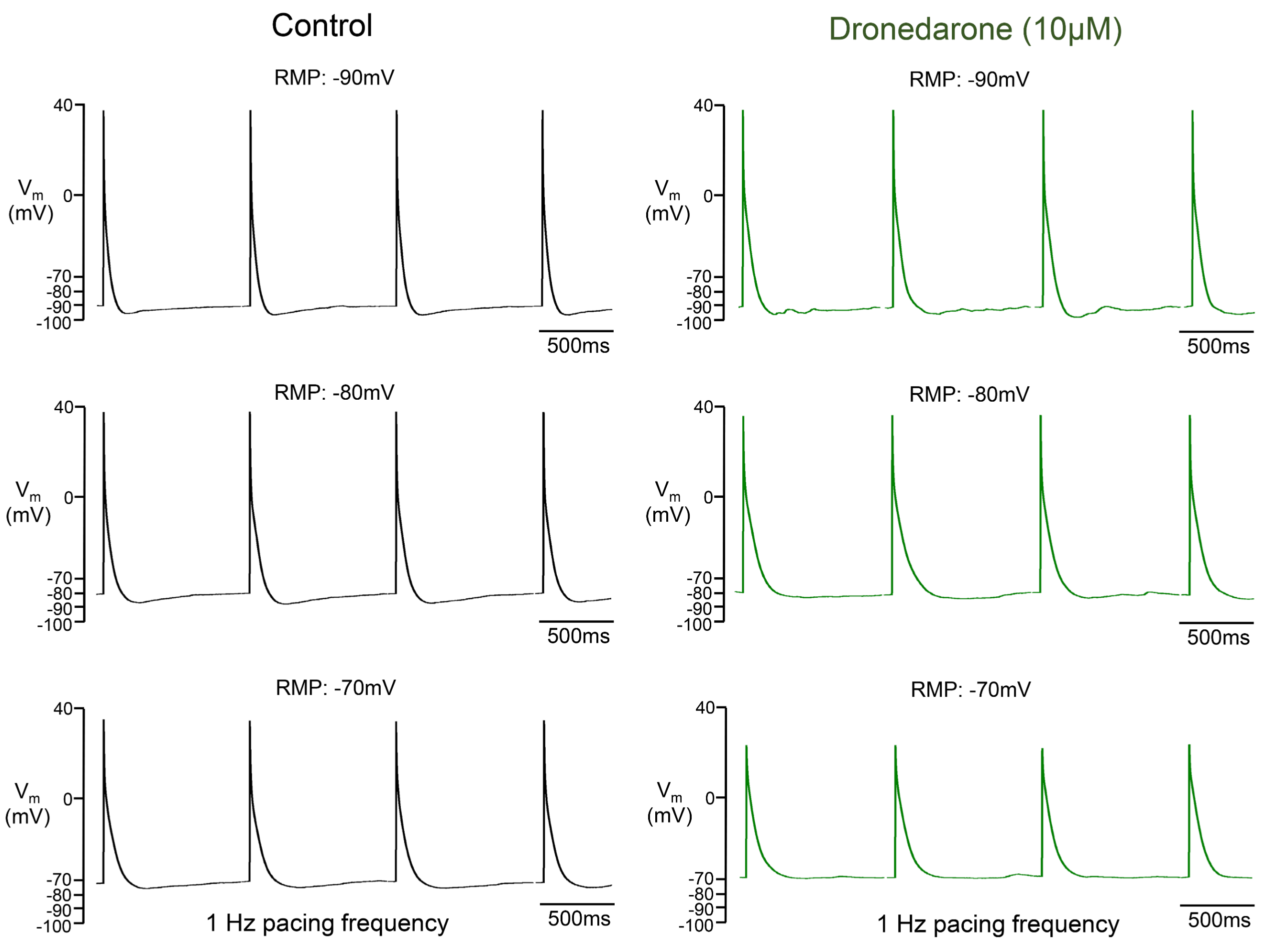

Supplementary Figure 1. Dronedarone causes greater reductions in the action potential amplitude (APA) at more positive resting membrane potentials (RMPs), in human induced pluripotent stem cell derived cardiac myocytes (hiPSC-CMs)

Example trains of hiPSC-CM action potentials recorded at -90, -80 and -70mV RMPs in the presence and absence of dronedarone (10µM), paced at 1Hz. Dronedarone causes a more exaggerated reduction in the APA at more positive RMPs. All examples are recorded from the same cell. The data was acquired using Signal v6 (Cambridge Electronic Design, Cambridge, UK)- a sweep-based data acquisition software. To show continuous traces the data has been transferred into Spike2 v7 (Cambridge Electronic Design, Cambridge, UK).

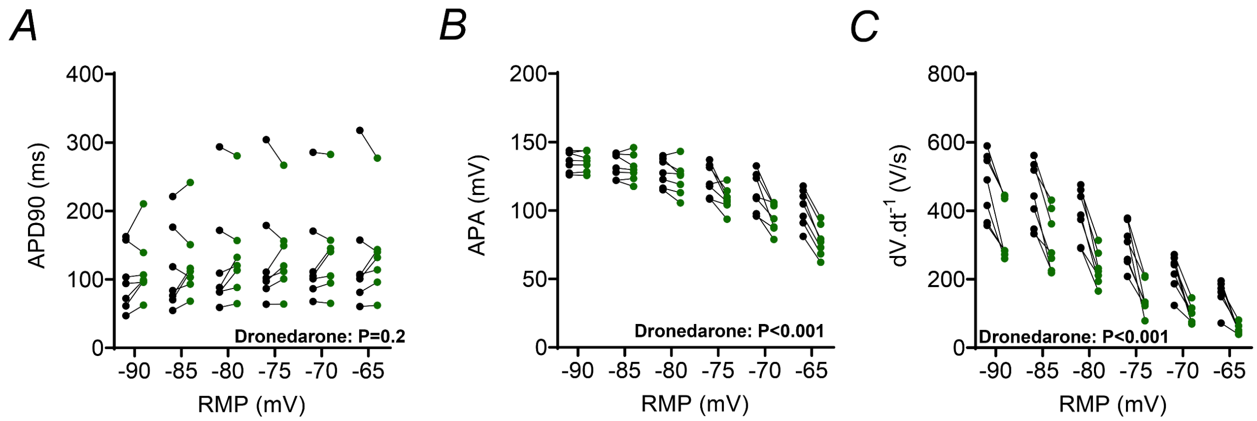

Supplementary Figure 2. Effects of dronedarone on human induced pluripotent stem cell derived cardiac myocytes (hiPSC-CM) action potentials

(A-C) Impact of dronedarone on action potential duration (APD90), action potential amplitude (APA) and AP peak upstroke velocity ($dV.dt^{-1}$), recorded over a range of resting membrane potentials (RMP; -90 to -65mV) respectively. hiPSC-CMs were paced at 1 Hz. All individual paired responses are shown at each RMP (N=7 myocytes).

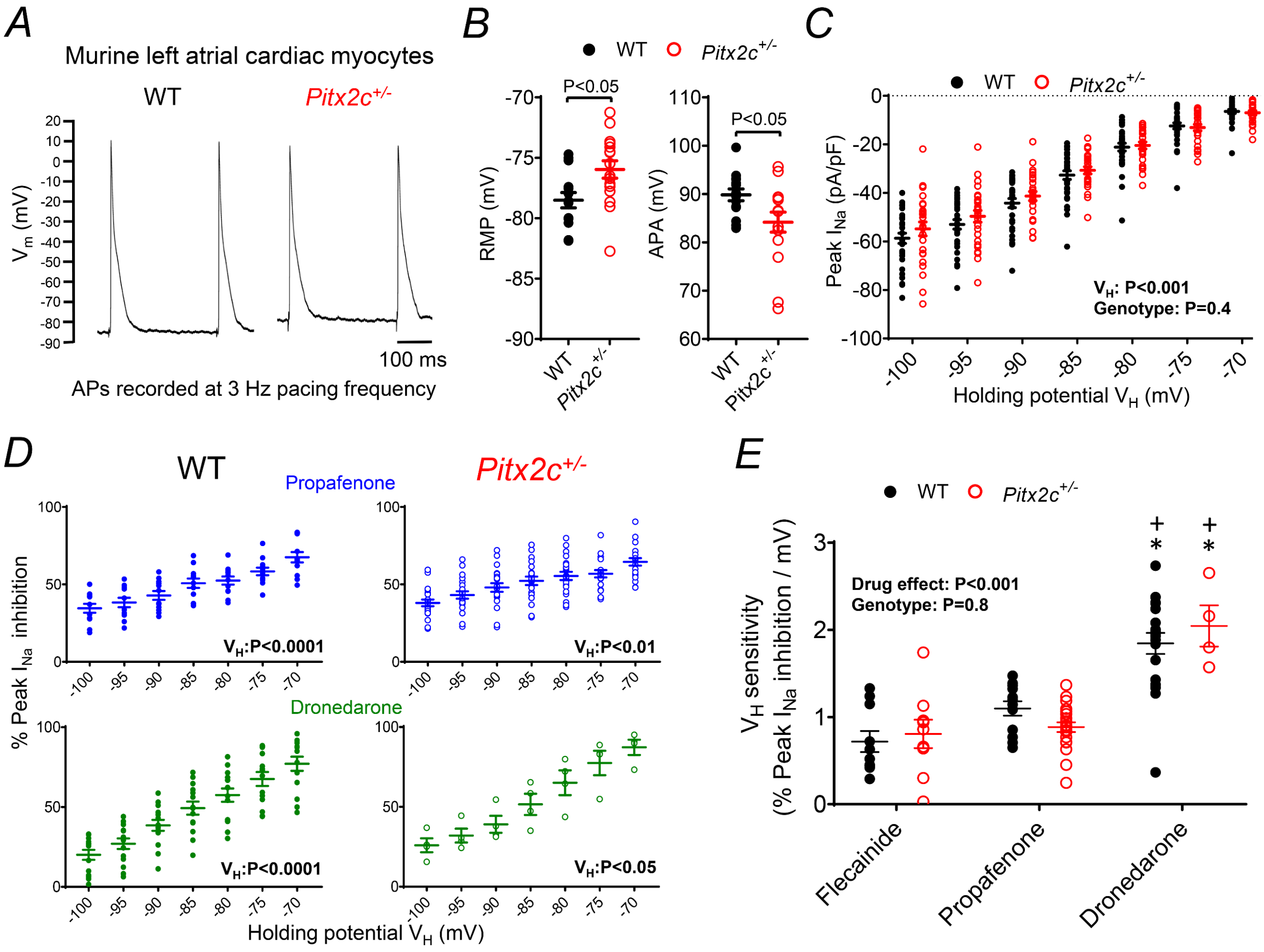

Supplementary Figure 3. Murine left atrial (LA) cardiac myocytes with reduced *Pitx2c* have a more positive resting membrane potential (RMP) and reduced action potential amplitude (APA) without depletion of native sodium currents (I_{Na}).

(A) Example microelectrode transmembrane action potentials (TAPs) recorded at 3Hz frequency from WT and *Pitx2c*$^{+/-}$ intact LA. **(B)** Mean RMP and APA for WT (N=14 cells, 5 animals) and *Pitx2c*$^{+/-}$ (N=16 cells, 5 animals). Data presented as mean±SEM, unpaired t-test. **(C)** Mean sodium currents (I_{Na}) recorded at different RMPs/holding potentials (V_H) in primary dissociated LA cardiac myocytes from WT (N=32 cells, 12 animals) and *Pitx2c*$^{+/-}$ (N=27 cells, 9 animals). Data presented as mean±SEM, 2-way repeated measures ANOVA. **(D)** Mean data from dissociated murine LA cardiac myocytes demonstrating the effect of RMP/V_H on I_{Na} inhibition by propafenone (300nM) and dronedarone (5µM) for WT and *Pitx2c*$^{+/-}$. **(E)** Comparison of the RMP/V_H sensitivities of flecainide (1µM), propafenone

(300nM) and dronedarone (5μM), calculated as the % increase in I_{Na} inhibition per mV increase in RMP/V_H in WT (N=41 cells, 17 animals) and *Pitx2c*$^{+/-}$ (N=34 cells, 14 animals). Data presented as mean±SEM. * denotes P<0.05 vs flecainide and + P<0.05 vs propafenone, two-way ANOVA with Bonferroni post hoc analysis.

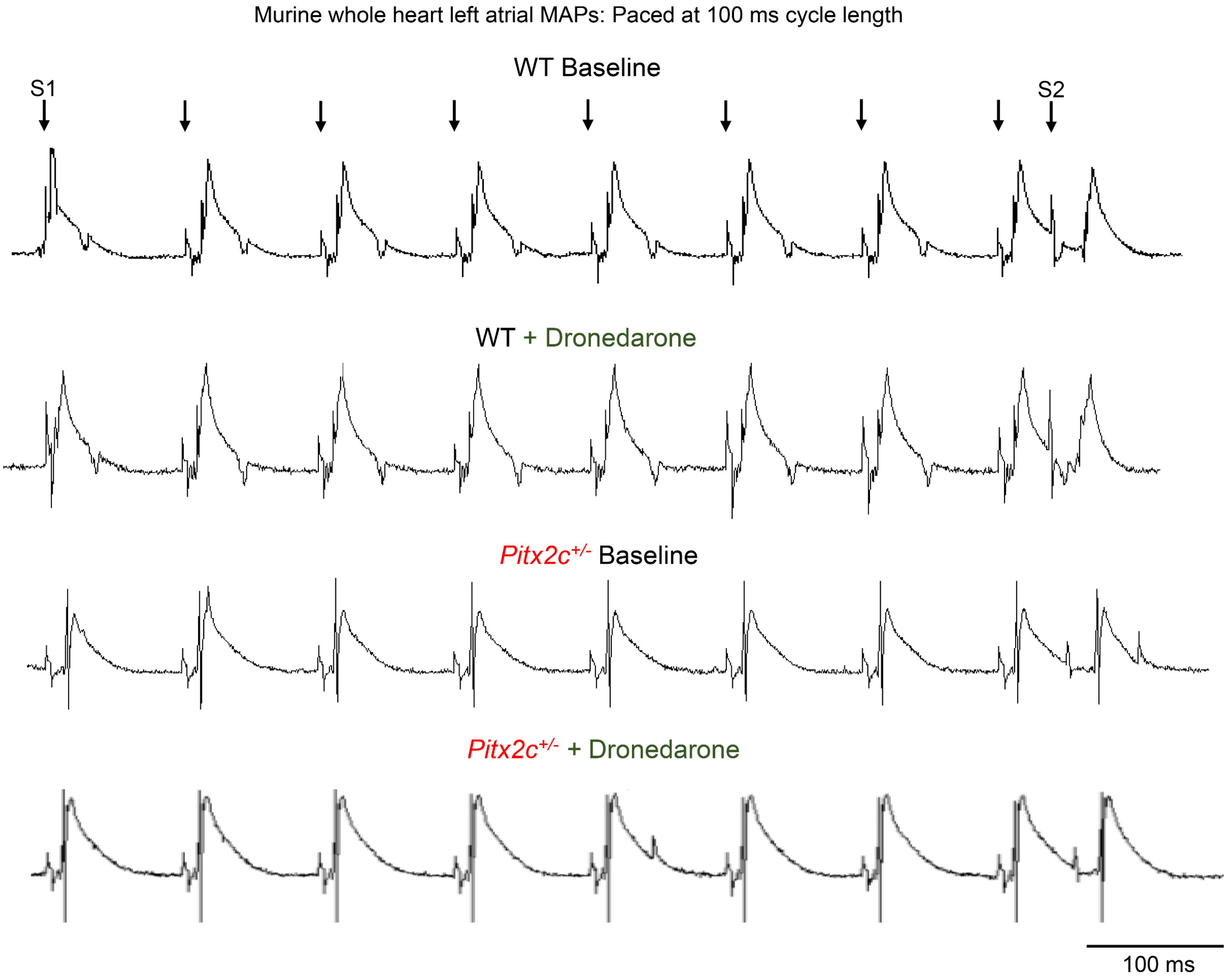

Supplementary Figure 4. Dronedarone causes greater effective refractory period (ERP) lengthening in murine left atria (LA) with reduced *Pitx2c* compared to wildtype. Example trains of LA monophasic action potentials (MAPs) recorded in WT and *Pitx2c*$^{+/-}$ hearts in presence and absence of dronedarone (10μM), at 100 ms pacing cycle length. The ERP was determined by using single premature extrastimuli (S2) after a train of 8 x S1 pacing, in 1ms decrements. For each trace the final premature S2 stimulated MAP is shown immediately before loss of capture. This example shows how dronedarone prolongs the ERP in *Pitx2c*$^{+/-}$ but not WT LA.

Supplementary References

1. Kirchhof P, Kahr PC, Kaese S, et al. PITX2c is expressed in the adult left atrium, and reducing Pitx2c expression promotes atrial fibrillation inducibility and complex changes in gene expression. Circulation Cardiovascular genetics Apr 2011;4:123-133.
2. Ackers-Johnson M, Li PY, Holmes AP, O'Brien SM, Pavlovic D, Foo RS. A Simplified, Langendorff-Free Method for Concomitant Isolation of Viable Cardiac Myocytes and Nonmyocytes From the Adult Mouse Heart. Circulation research Sep 30 2016;119:909-920.
3. Syeda F, Holmes AP, Yu TY, et al. PITX2 Modulates Atrial Membrane Potential and the Antiarrhythmic Effects of Sodium-Channel Blockers. Journal of the American College of Cardiology Oct 25 2016;68:1881-1894.
4. Kirchhof P, Benussi S, Kotecha D, et al. 2016 ESC Guidelines for the management of atrial fibrillation developed in collaboration with EACTS. European heart journal Oct 2016;37:2893-2962T.
5. Gupta S, Manchanda R. A computational model of large conductance voltage and calcium activated potassium channels: implications for calcium dynamics and electrophysiology in detrusor smooth muscle cells. J Comput Neurosci Jun 2019;46:233-256.
6. O'Shea C, Holmes AP, Yu TY, et al. ElectroMap: High-throughput open-source software for analysis and mapping of cardiac electrophysiology. Sci Rep Feb 4 2019;9:1389.
7. Workman AJ, Kane KA, Rankin AC. The contribution of ionic currents to changes in refractoriness of human atrial myocytes associated with chronic atrial fibrillation. Cardiovascular research Nov 2001;52:226-235.

8. Workman AJ, Pau D, Redpath CJ, Marshall GE, Russell JA, Norrie J, Kane KA, Rankin AC. Atrial cellular electrophysiological changes in patients with ventricular dysfunction may predispose to AF. Heart rhythm : the official journal of the Heart Rhythm Society Apr 2009;6:445-451.

9. Holmes AP, Yu TY, Tull S, Syeda F, Kuhlmann SM, O'Brien S-M, Patel P, Brain KL, Pavlovic D, Brown NA, Fabritz L, Kirchhof P. A Regional Reduction in Ito and IKACh in the Murine Posterior Left Atrial Myocardium Is Associated with Action Potential Prolongation and Increased Ectopic Activity. PloS one 2016 2016;11:e0154077.

10. Yu TY, Syeda F, Holmes AP, Osborne B, Dehghani H, Brain KL, Kirchhof P, Fabritz L. An automated system using spatial oversampling for optical mapping in murine atria. Development and validation with monophasic and transmembrane action potentials. Progress in biophysics and molecular biology Aug 15 2014.
